# Book Reviews

**Published:** 1802-01

**Authors:** 


### CRITICAL RETROSPECT

OF

### MEDICAL AND PHYSICAL LITERATURE.

### [ FOREIGN AND DOMESTIC. ]

---

*Versuch*, &c. i. e. Eſſay, theoretical and practical, on the Effects of Medicines, by *Frederic Kretschmar*, vol. i. pp. 216; vol. ii. pp. 528. 8vo. 1800. *Halle*, for *Hemmerce* and *Schwetshke*.

NOTWITHSTANDING the improvements and diſcoveries which have been lately made by phyſiologiſts and chemiſts in the knowledge of the virtues of medicines, and of human organization, we are ſtill far from being able to eſtabliſh a perfect ſyſtem of materia medica, or to comprehend the actions of remedies under a complete and perfect theory. Conſcious of this circumſtance, the author profeſſes his work to be nothing but a previous eſſay, on which he intends to conſtruct, in future, a completer work on the ſame ſubject; it contains, however, ſeveral intereſting hints for the practitioner, and uſeful materials for the completion of a greater work of that kind. With reſpect to the principles on which the reſearches in this work are founded, the author does not adhere to any particular ſyſtem now in vogue, but has choſen from all what he thought beſt and moſt proper. In the firſt part, he treats of the following ſubjects. *Chap. I.* Determination of the difference between medicines, poiſons, and nutriments. *Chap. II.* Manner in which medicines are communicated to the living body, and on the organs which are firſt acted upon. *Chap. III.* Laws, according to which the actions of medicines proceed. *Sect.* i. Action of medicines according to phyſical laws. *Sect.* ii. Action of medicines according to organic laws. *Sect.* iii. Action of medicines according to organic vital laws. *Sect.* iv. Action of medicines according to vital laws, or according to the laws of the vis vitalis. *Sect.* v. Action with reſpect to the reaction of ſyſtems and organs, to which he refers the laws of aſſociation and antagony of ſympathy and antipathy. The contents of the ſecond part are the following. *Chap. I.* Introduction and plan for this eſſay of a ſpecial materia medica. *Sect.* i. Reſults of experience. *Sect.* ii. Action of medicines. Analogy of action, chemical and perceptible by the ſenſes. *Chap. II.* Survey of the ſpecial or compound effects of medicines. *Sect.* i. On the emetic effect. *Sect.* ii. On the purgative effect. *Sect.* iii. On the exciting, heating, diaphoretic, and cooling effects. *Sect.* iv. Summary ſurvey of the actions of medicines on abſorption and ſecretion. *Sect.* v. On the action of abſorbing acidities. *Sect.* vi. On carminatives; and *Sect.* vii. On the anthelmintic action. *Chap. III.* Survey of the

ſpecial

ſpecial action of remedies, according to their perceptible and chemical effects. 1. On the narcotic principle of vegetables. 2. On the aromatic principle. 3. On the volatile acrid principle. 4. On the bitter principle of vegetables. 5. On the aſtringent principle. 6. On acids. 7. On vegetable mucilage, gums, ſtarch, gluten, ſugar, groſs oils, &c. 8. On reſins. 9. Eſſential oils, camphor. 10. On ſpices. The work concludes with an apparatus *medicaminum*.

---

*Medicinisch Chirurgische Bemerkungen*, i. e. Medical and Surgical Obſervations, by Chr. F. Schumacher, Profeſſor at Copenhagen, &c. Vol. 1, 1800, large 8vo. pp. 22. Copenhagen, Brummer.

The author purpoſes to publiſh by degrees, the more remarkable caſes of his Hoſpital practice; and in order to enable the reader to compare more properly the courſe and ſymptoms of the diſeaſe with the effects of the remedies that have been employed, he communicates a ſeries of caſes of each principal diſeaſe. The remedies that are made uſe of are the moſt ſimple and the leaſt expenſive. When emetics are indicated, he uſes, inſtead of ipecacuanha, 15 grains of gratiola, ſometimes mixed with 5 grains of rhubarb. In the gangrene, he applies, inſtead of the bark, the cortex Hippocaſtani and ſalic; inſtead of ſarſaparilla he gives the carex arenaria. In intermittent fevers he has ſucceſsfully employed the cortex Soimydæ; in the fluor albus he found the faba pichurim, half a drachm pro doſe for three times a day, very efficacious. For bliſters he recommends a mixture of wax, turpentine, and the powder of Spaniſh flies. In contuſions, equal parts of vinegar and water are ſerviceable. After conſiderable wounds or operations, he gives a ſimple tincture of opium every two or three hours, conſiſting of op. crud. dr. j. ſpir. vin. rectific. unc. j. He employed the cortex caribæus in intermittent fevers without ſucceſs, but with ſome uſe in the lumbago; he never remarked any emetic quality of that remedy during its uſe. Such, and ſimilar remarks, are contained in this work, which deſerves the attention of hoſpital phyſicians, and of thoſe who are obliged to have recourſe to cheap and ſimple remedies. The greateſt part of this volume, however, is devoted to the leſions of the head, of which 217 caſes fell under the author's care, from the 1ſt. of November 1795, to the 1ſt. of March 1800, 127 of which ended fatally. The method of treatment conſiſts of cold fomentations on the ſhaved head, of veneſection, leeches, and the internal uſe of camphor; but he recommends, above every thing, the timely application of cold fomentations, of the uſe of which he is ſo much perſuaded, that in moſt caſes the fatal event might be attributed to their being neglected; but they ought always to be kept properly cold, by the addition of ice, ſnow, &c. Theſe are the contents of this uſeful publication, the continuation of which we heartily approve.

---

*Observations on the Cancerous Breast; consisting chiefly of original Correspondence between the Author and Dr. Baillie, Mr. Cline, Dr. Babington, Mr. Abernethy, and Dr. Stokes; published by Permission of the Writers; with an introductory Letter to Mr. Pitcairn. By* Joseph Adams, M. D. *of the Royal College of Physicians, and Physician in the Island of Madeira.* 8vo. pp. 115, Longman and Rees.

The Author of the work before us has been favourably known to the public as an acute reaſoner, an able controverſialiſt, and a very zealous defender of thoſe theoretical ſpeculations on various diſeaſes, which proclaimed the genius of the late John Hunter.

In his "Obſervations on Morbid Poiſons," (publiſhed in 1795,) Dr. Adams has taken great pains to eſtabliſh a very ſingular opinion concerning the origin of *carcinoma,* which had been merely hinted at by Dr. Hunter, and which is again urſued at conſiderable length in the treatiſe before us.

There are few diſeaſes in which the utmoſt length of conjecture, and the greateſt latitude of experiment, are more to be allowed and encouraged than in cancer.

A melancholy conviction of the extent of its ravages, and of the inability of art to check it progreſs by any certain rules, has been lately evinced by an inſtitution in this metropolis, for the expreſs purpoſe of inveſtigating the nature of this dreadful calamity; and we feel diſpoſed to give a conſiderable ſhare of attention to the conjectures advanced by the ingenious author of the preſent publication. As this is to be conſidered as a ſequel to the chapter in the *Obſervations on Morbid Poiſons,* juſt alluded to, we ſhall beg to preſent our Readers with the ſum and ſubſtance of it in as few words as poſſible.

Schirrus, Dr. A. obſerves, or the early ſtage of carcinoma, is always compoſed of certain *cysts,* which, he thinks, conſtitute its true character, and which are filled with fluids of different complections. Mr. Hunter has ſpoken of the cyſts as being *cancerous hydatids,* and this is the opinion which Dr. A. follows up in his ſubſequent obſervations, and which conſtitutes their originality. Theſe cyſts have ſome peculiarites in their appearance and progreſs; the one, that their increaſe is not towards the ſurface, like matter in a common abſceſs, but in every direction; another peculiarity in the diſeaſe is a diſpoſition to fungate even before the ſkin is broken, and in a very high degree after it has become an open ſore. After this period the progreſs of the diſeaſe is uncertain, ſometimes with a rapid ulceration of the fungus, which, in ſloughing away, expoſes ſeveral roundiſh *foveoli*, which are the cyſts with their contents, changed from their original appearance to that of an opake pale, reſembling half diſſolved leather. This alternate progreſs, namely, of the extenſion of theſe cyſts, of the formation of fungus around them, of the ſloughing of this fungus and the conſequent diſcharge of the contents of the cyſts, and of the attempts at granulating mulating which are made by the furrounding parts, forms the principal phenomena of this difeafe.

We fhall not further purfue the arguments of the Author in his Obfervations on Morbid Poifons, in order to eftablifh his opinion on the origin of cancer; but he concludes the fubject by propofing a fet of queries, wherein are the following: Is the fimple hydatid the firft form of carcinoma? Have thefe hydatids a life independent of the fubject in which they grow, excepting as parafites? &c. &c.

The anfwer to feveral of thefe queries is given by the Author in the work which we are now to confider, and to which it is high time to revert.

This volume confifts of correfpondence between the Author and Dr. Baillie, Mr. Cline, Dr. Babington, Mr. Abernethy, and Dr. Stokes; in which Dr. A. ftates pretty fully his peculiar opinions, and his correfpondents fome of the objections which may be urged againft them, and other very valuable illuftrative facts. Perhaps it would have affifted the reader in forming a clearer idea of the Author's theory, if the facts on which it is founded had not been prefented in fo defultory a form; but the authenticity which is attached to original correfpondence may make amends for this defect.

Almoft the whole of our knowledge on that curious parafitical animal, the human Hydatid, is contained in an excellant paper by Dr. J. Hunter, inferted in the *Transactions of a Society for improving Medical and Chirurgical Knowledge*, which the author has prefixed entire to the fubject matter of this work. Dr. Hunter's paper confifts of the hiftory of a diffection of a perfon who died with fymptoms of difeafed bladder, in which vifcus was contained a large number of thefe fingular animals, of a fize varying from an inch and a half in diameter to that of a pin's head.

The ftructure of the hydatid is moft clearly defcribed by Dr. Hunter in the following words:

" As to the ftructure of the hydatids, it was the fame in large and fmall; a tranfparent bag, uniformly round and fmooth, filled with clear water. The bag appeared to confift of two coats, or layers; for, on handling them, the outer coat would get rumpled, and occafion a degree of opacity, but by wiping the hydatid it became again clear and tranfparent. They appeared to be completely fpherical, except that the large ones were a little flattened by their own weight when laid on a plate. They adhered no where to the fides of the fac, nor to one another. When they were opened, their coats poffeffed a ftrong contractile force, fo as to roll themfelves up in part. On examining a number of hydatids, fome of them appeared of an amber colour, and with thicker coats than the reft; and when opened, their inner furface was found covered with fmall hydatids, which were not fo large as the heads of pins, and looked like minute pearls or ftuds fet in the inner coat.

" Some of the water containing the fmall grains mentioned above, was examined with a microfcope, and found to have floating in it numerous minute hydatids, of which the largeft were the little grains visible to the naked eye, and a two hundredth part of an inch in diameter; the smallest were less than a red globule of blood, and they were of all intermediate sizes. The coats of the largest were a little rough with numerous filaments, or *villi*; and on using a deeper magnifier they had somewhat of a mulberry appearance.

"When the young ones growing in the coats of the larger were examined with the microscope, they were found not to be set in the coats, like pearls, but to be covered by a thin transparent membrane, so as to lie between two layers. It is not improbable that the small globules attach themselves by the *villi* to the side of the hydatid and to each other, and thereby give the appearance of being covered by a thin membrane. However that may be, the globules being found of various sizes floating in the liquor, seems to prove that they are originally formed there, and not in the coats of the hydatid, upon which they are afterwards deposited. The number of those that had young ones in them, was few in proportion to the others.

"The hydatids in their growth and decay appear to pass through various stages; they are first found floating in the fluid that fills the hydatid, and afterwards attached to its coats. The hydatid thus pregnant with young, if the expression may be allowed, adheres to the neighbouring parts, increases in size, and becomes itself a sac, containing numerous small hydatids. These, after a certain time, decay, and the skins or empty bags are squeezed together into a substance like isinglass. It is probable they still undergo a further change; two small bodies of the size of the common bean, of a cheese-like consistence, and covered with a skin, were taken notice of adhering to the bladder, near its neck; it may be a question whether those were not the remains of hydatids? but that must be determined by future observations."

The hydatids of sheep differ from the human hydatid, in possessing besides a more muscular bag filled with a transparent liquor, a mouth or longitudinal aperture, and a neck composed of rings. These hydatids are contained in the abdomen of the animal; others are sometimes found in the brain, which again want the mouth and neck above described, and give the disease called the *staggers*. Each kind of hydatid, when put in warm water, moves about briskly, with a kind of peristaltic motion of the whole body, though it has been taken out of the sheep for several hours.

The first letter contained in this volume is addressed to James Pitcairn, Esq. in which Dr. A. makes some observations on the structure of the hydatid, and observes, very justly, that "the most "simple idea of animal life we can well form, is that of the hydatid, consisting only of a membranous bag containing a transparent fluid. Those in the human body give no other proofs of "life than a contractile power."

The second letter is from the author to Dr. Baillie. Dr. A. first feelingly laments his deprivation of those opportunites for improvement which London affords to the anatomist. "At a distance from science,

ſcience, from hoſpitals, from diſſecting rooms, and often from books, to be thus remembered by thoſe who luxuriate in all theſe delights, may, for a time, renew thoſe ſcenes we have reluctantly left, and make us anxious to protract the charming illuſion." He alſo candidly acknowledges the defects of his former work, the Treatiſe on Morbid Poiſons.

The third letter is an anſwer from Dr. Baillie to the author, and we cannot refuſe to give our readers the following accurate and valuable remarks which it contains on the ſtructure of ſchirrus. Dr. B. obſerves, "In parts which have become ſcirrhous, I have commonly obſerved the ſtructure to conſiſt of a very firm light brown ſubſtance, interſected by membranous or ligamentous ſepta, which run in various directions. The membranous ſepta are more numerous, and of greater thickneſs in ſome caſes than in others. There is occaſionally mixed with this ſtructure a cartilaginous ſubſtance. The whole ſtructure I have ſometimes known to be cartilaginous, reſembling very much a piece of common cartilage, which had been previouſly rendered ſoft by being ſteeped for ſome time in a diſſolving fluid.

"Ulcers are often formed in ſcirrhous ſtructures, and fungous excreſcencies occaſionally grow from them. Cyſts containing a kind of ſerous fluid are ſometimes found in ſcirrhous ſtructure; but they ſeem to me frequently wanting. They occur, I believe, moſt commonly in the breaſt and teſticle, and theſe glands in a ſcirrhous ſtate I have had few opportunities of examining. From what I have obſerved, I ſhould be inclined to believe, that cyſts are only ſometimes formed in a ſcirrhous ſtructure, but are not eſſential to it. In this, however, I may be miſtaken; and it may be found by a more minute obſervation, that the formation of cyſts always conſtitutes a part of a ſcirrhous ſtructure. If you ſhould be able to eſtabliſh this or any other general obſervation about the nature of ſcirrhus, it will give me real ſatisfaction.

"I have known a ſubſtance which poſſeſſed the common characters of ſcirrhous ſtructure to be converted into a kind of bony matter. In this, I believe, that the earthy part will be generally found to be in a larger proportion to the animal part than in common bone. Muſcular and membranous parts I have known to be affected with ſcirrhus, as well as thoſe which are ſtrictly glandular. A fatty membrane I have ſeen affected with the ſame diſeaſe. The fat was almoſt as hard as a piece of griſtle."

In the fourth letter, (from the author to Dr. Baillie) he explains more at large his opinion on the nature of hydatid, and the proofs of its exiſtence in carcinoma. In this part of the ſubject the author is ſo clear and preciſe, that our readers cannot form a better idea of it than in his own words. "By the term hydatid," he obſerves, "I mean an animal, conſiſting only of a cyſt and its contents, incapable of exiſting but in living animal matter, having the powers of ſecreting or abſorbing from its nidus the food which fills its whole cavity, and of producing an offspring ſimilar to itſelf by no generative organs that can be traced. If it ſhould

ſhould be objected that the etymology of the word would confine it to thoſe cyſts which contain a watery fluid, I would remark, that ſuch is not the caſe with the common hydatid, the contents of which are coagulable, and ſometimes tinged with red particles of blood. For this and other reaſons, I ſhould divide theſe into hydatis *lymphatica*, and hydatis *cruenta*."

"That which is the ſubject of our preſent enquiry, I ſhould call hydatis *carcinomatoſa*, which, beſides the difference of its contents, has alſo the property of ſtimulating the part in which it lives to form a kind of fungus, for purpoſes I ſhall endeavour hereafter to point out: my firſt buſineſs is to prove, if poſſible, the animalcular exiſtence of carcinoma; for this fungus, though in the cancerous breaſt it is uſually conſidered as the whole of the ſcirrhus, appears to me only an appendage to the carcinomatous hydatid."

"As you have been leſs converſant with the cancer in the breaſt than the viſcera, you may, perhaps, not have examined with your uſual accuracy a quantity of apparently diſeaſed fat; which, when the part is very much enlarged, is found incloſed in different portions of the ſcirrhus, and of which the whole poſterior part of the tumour, as well as the ſpace from that to the axilla is uſually made up. This diſeaſed fat, as it is ſometimes called from its appearing thinner, more tranſparent, and of a greeniſh yellow hue, I have found to have all the properties aſcribed to the hydatid in the human ſubject."

Dr. A. then reverts to the proofs of the ſeparate exiſtence of the common hydatid given in Dr. Baillie's Morbid Anatomy. The chief is, that the hydatid of the ſheep, when taken out of the body and put into warm water, has been ſeen to move. This, indeed, has not been obſerved with the hydatid of the human liver; but as Dr. B. obſerves, the reaſon may be that the body is ſeldom examined till many hours after death. In the caſe given by Dr. Hunter, which we have juſt alluded to, he found likewiſe that the hydatids when opened, retained a ſtrong *contractile force* ſo as to roll themſelves up in part. This power of contraction, Dr. Adams obſerves, if not dependant on elaſticity, is a ſufficient proof of the life of theſe hydatids. If, therefore, the ſame property, unconnected with elaſticity, be found in the cancerous breaſt, it will be ſufficient (Dr. A. imagines) to eſtabliſh the point of the hydatid-life of this diſeaſed part. This he demonſtrates in the following manner.

"Immediately after the operation, take the amputated part, and cut it in a tranverſe, or indeed in any direction, and wherever you diſcover this fatty appearance, you will ſee the ſurface at firſt ſmooth under your knife. In an inſtant after, you will find a papillary appearance all over the yellow-green ſurface. Each of theſe papillæ you will find to be part of the contents of a capſule, the contraction of which has produced this conical figure. If the amputated part has been expoſed long to the cold, or thrown into water and left there for ſeveral hours, a ſection of it no longer exhibits exhibits this appearance, nor is it poffible in this ftage to diftinguifh at firft fight what I call carcinomatous hydatids from common fat. By this it appears that the yellow-green tranfparency of the fat, and the contractile power of the inclofing tunics could only arife from life, and the degree of heat with which that life was attended."

The next point on which Dr. A. exercifes his conjectures, is on the ufe of the formation of cartilage before ulceration, and of the fungus after it, which fpring up round the ulcer; calling in aid of his explanation that principle fo well laid down by Hunter, that living animals lodged within another living body, do not whilft alive ftimulate to fuppuration the parts which form their nidus; but that when dead they act as mere extraneous matter, and caufe this procefs to take place: Dr. A. thence explains the ufe of the carcinomatous fungus in the following terms:

"Now, if carcinomata pafs through the fame ftages as Dr. J. Hunter has remarked of the common or lympahtic hydatid, is it not probable that on the death of any of them fuppuration will follow, and that this fuppuration may expofe the living hydatids in fuch a manner that many of them may die from not being furrounded by living animal matter? To prevent this, I conceive a fungus is formed, which inclofes individuals or clufters of them in feparate compartments, fo that the death of one fet produces no effect on the reft."

"As far as my obfervation extends, this fungus grows in every direction where it is neceffary to preferve the hydatids. When a clufter of hydatids dies, the fungus between it and the furface ulcerates, or floughs flowly, till the compartment containing them is expofed. By this time, if the progrefs has been very flow, all the tunics of the hydatids are detached, and the furface being clean will make an attempt at healing. If no dead hydatids are in the neighbourhood, it will often, for a time, fcab, or even fkin over. But if, when the cavity is expofed, fome of the tunics of the hydatids retain their attachments, the attempt at healing will only produce an exuberance of fungus with retorted edges. This will continue till all the tunics or fragments of them are detached; after which, if no new impediment arifes, the edges will take a different direction, and the part heal for a time."

The fifth letter, (from Mr. Cline to the author). The writer obferves, that he has often met with the cells in cancerous tumours, containing a greenifh yellow liquor, as mentioned by Dr. A. but they did not give Mr. C. the idea of being living hydatids. The writer alfo gives the following valuable practical directions concerning the removal of encyfted tumours.

"When an encyfted tumour is fmall, it may be cured by an opening that will difcharge its contents; and then by applying lapis fepticus to the internal furface, fo as to deftroy the cyft. But when they are very large, this practice muft be dangerous, becaufe the fuppurating furface might be greater than the conftitution is able to fupport. The removal or deftruction of the cyft is generally generally neceffary, for it rarely has any difpofition to heal. Even the leaving a very fmall part of the cyft will be fufficient to prevent the complete healing of the wound."

In the fucceeding letter, (in anfwer to the former,) Dr. Adams endeavours to explain the nature of the various cavities or cyfts connected with carcinoma. He fuppofes three kinds. The firft is the common hydatid, in which, after the contents have been wafhed away, on throwing the amputated part in water, the cyft ftill preferves its figure, and remains an empty cavity, or only filled with ferum. The fecond kind of cyft is a cavity filled with a gelatinous fubftance, which the author fuppofes to be carcinomatous hydatids, which have gone through their various ftages of birth, growth, and decay, and are retained in the enclofed fungus. A third fpecies is a cavity compofed of cells, filled with a dark bloody fluid, to which the author gives the appellation of *Hydatis Cruenta*.

In the eighth letter, (which is from the author to Mr. Abernethy,) Dr. A. explains more at large the final caufe of the generation of fungus. This is in a great meafure a repetition of what we have given our readers from the fourth letter; but the following paffages will include all the moft material fubftance of the author's hypothefis, which therefore we fhall quote.

"In examining a carcinomatous breaft amputated in an early period, we meet with little or no fungus. By an early ftage I mean, before the difeafe, how long foever it may have exifted, has made any confiderable progrefs. If the progrefs has been fufficient to exhibit any fuperficial marks, by a circumfcribed puckering of the fkin, we find the fungus ufually confined to the fpace between the carcinomatous hydatids and the furface; but if the difeafe has made confiderable progrefs, fo that the whole breaft is much enlarged, it is then that we find various compartments in the fungus filled with hydatids in different ftates of their progrefs towards maturity and death.

"Hence it feems as if the hydatids had a period of exiftence fhort in proportion as their powers of multiplication are greater. Till they multiply (fuppofing them in a fituation that affords them a nidus for it) they appear more or lefs in a torpid ftate, occafionally growing, and at other times ftationary. But the death, or perhaps even the approach towards death, of any individual or number of carcinomatous hydatids, inftantly becomes a ftimulus to the furrounding parts to generate this fungus, which, by feparating the dead from the living, produces in different parts of the fame breaft two different actions at the fame time. One is a kind of ulceration, or more commonly continual floughing of the fungus which inclofes the dead hydatids; the other is the formation of new fungus to protect the living hydatid, and in many inftances, if not in all, the fungus becomes itfelf a nidus for the generation as well as protection of future hydatids. That it does fo for hydatis cruenta, when fuch are the contents of a cancerous breaft, we have every proof that our fenfes can furnifh. For in thefe cafes the the fungus is always much fofter and fpreads fafter, if the integuments are removed by the knife, cauftic, or ulceration, and the whole appearance when removed is fimilar to the defcription of thofe hydatids which have efcaped from the uterus, adhering to a fpongy fubftance refembling or ferving as a placenta."

[ To be continued. ]

---

*Obfervations on the Opinion of Dr. Langslow, that Extravasation is the general Cause of Apoplexy, in Letters to a young Surgeon*; by WILLIAM CROWFOOT. 8vo. pp. 46. Robinfons. London, Oct. 31, 1801.

IN a private letter to the Editors, Mr. C. complains that his Statement, which appeared in our laft Number "was not intended to be obtruded upon the public in fo crude and incomplete a form, nor with the verbal errors it contains. He does not think his opponent juftifiable in fending what was intended for a fmall circle, before the public at large, without the knowledge and confent of the writer." In the prefent pamphlet, Mr. C. has given that Statement of the Cafe which he wifhes to be confidered the only one intended by him for general infpection. It does not differ materially in facts or opinions from that given in the Journal. A fecond Cafe is added, very fimilar to the firft, which was attended by Mr. C's friend, Mr. Davey, who gives the following relation of it.

"When I firft faw her, which was not lefs than two hours and a half from the commencement of the attack, fhe was juft recovered from a fit, which, from its fymptoms, (defcribed to me by the perfons prefent) I judged to be apoplectic, and of that kind which is termed by writers of the moft refpectable authority, the nervous apoplexy.

"She was now perfectly collected in her fenfes, but had a difficulty in articulation, with a want of recollection. To explain which I muft obferve, her ideas of things were perfectly accurate, though fhe had forgotten the words which expreffed them; I fay collected, becaufe when fhe was at a lofs for a proper word, if you mentioned it, fhe directly ufed it.

"She complained of head-ach, ficknefs, naufea, numbnefs all over her right fide, with want of power to move that arm, thigh and leg, without the greateft exertion, and then could merely drag them after her. For fome days previous to this attack fhe had been troubled with vertigo, naufea, general uneafinefs, pain in the head, and drowfinefs. The bowels had been regular.

"As Dr. Duncan, in his lectures on cafes like this, where evidently no extravafation was prefent, recommends to his pupils to bleed in a very fmall quantity, to avoid cenfure from not doing that which long cuftom has eftablifhed, (although he thinks it detrimental to his patient rather than the contrary): I thought it prudent to follow fuch high authority, (though contrary to my own opinion) and took away not more than three ounces of blood, juft to fay fhe was let blood, and ordered an emetic to be given immediately, together with a bliſter to the back. After the operation of the emetic, a ſtimulating purgative mixture (ſo as to evacuate the contents of the bowels, and remove any thing offenſive which might be lodged there) was ordered, and likewiſe a rubifacient embrocation for the part affected.

" I ſaw the patient no more, but was happy to hear from my friend, Mr. C. that moſt complete ſucceſs had attended this mode of treatment.

" H. S. D."

" In addition to this teſtimony, I can affirm, that on the following day the ſeverity of the attack had left threatening ſymptoms of hemiplegia, the right ſide being much affected, and which did not perfectly recover for ſome days after."

Mr. C. appears to us to have adopted the opinions of Dr. Kirkland in too great an extent; whereas an author, like Dr. K. who deſigns to overturn old opinions and introduce new ones, always puſhes his peculiar notions too far, and ſhould be read with that impreſſion, and certain grains of allowance admitted accordingly. Dr. K. appears to ſtumble at the very threſhold of his Commentary, for he argues thus. I found two caſes of violent concuſſion of the brain by *mechanical* means, attended with *fracture* of the ſcull, which terminated fatally in a few days. After death, blood was found in contact with the brain. The patients had no other ſign or ſymptom of Apoplexy, except the ſudden falling down and afterwards dying: THEREFORE effuſion of blood within the cranium is *never the cauſe of true Apoplexy;* and "*erroneous* opinions have been drawn from appearances in the diſſection of dead bodies." p. 21. He then reviſes other ſimilar Caſes, determined to abide by this extraordinary inference, and attempts to eſtabliſh a diſtinction between Apoplexy and Coma, which, we fear, may lead to an erroneous and dangerous practice if generally adopted.

It is probable, however, that Mr. C. does not intend *jurare in verba magiſtri*, as the following paſſages will evince.

" I ſhall conſider the queſtion of extravaſation, &c. as the general cauſe of apoplexy, to be determined in my favour, until my opponent brings at leaſt equal evidence to invalidate what I have advanced. Suppoſing, therefore, this to be ſet at reſt, I proceed agreeably to my deſign, to the treatment of apoplexy, ſo far as regards the uſe of emetics, &c.

" For the cure of apoplexy ariſing from internal cauſes, Dr. Cullen recommends bleeding largely and purging; and he ſays, vomiting has been much employed by ſome practitioners and writers; but *apprehending* this might impel the blood with too much violence into the veſſels of the head, *I have never employed it.* This appears *to be all* Cullen ſays upon this ſubject, and it is very clear he is ſpeaking of a diſeaſe (coma) different from what we are now conſidering, and in which a judicious practitioner would heſitate to recommend ſuch a remedy.

" That emetics in apoplexy are neither new nor extraordinary, we learn by this extract from Cullen; and from my opponent's favourite, favourite, Brooks, we have the names of Sydenham, Boerhaave, Heifter, and Pitcairn, to fanction their ufe. But I do not rely upon them, becaufe large bleedings were generally prefcribed, and the practice, I have reafon to believe, was prejudicial to the patient, &c.

"I fhall now return to the main defign of this paper, the fubject of emetics. 'Allow me to afk,' fays the Doctor, 'how the brain can be oppreffed by a foul ftomach?' Without entering into any explanations as to the fympathy which is known to exift between the head and ftomach, I fhall briefly obferve, that no one I believe, except himfelf, would affert the brain was oppreffed, *or suffered compression,* when there was no other foundation for that opinion than pain in the head, flight ficknefs, and naufea?

"Again, 'Does Mr. C. really think that ftimulating the ftomach by emetics, will produce a freer circulation than emptying the blood veffels themfelves? And further, that plenitude is more expeditioufly obviated by vomiting than bleeding?' To thefe enquiries I anfwer, that *diftention* of the ftomach may affect the brain by preventing the free expanfion of the lungs, and of courfe a free return of the blood; and therefore, to remove this preffure from the diaphragm, will produce a freer circulation, and obviate that fullnefs more expeditioufly by vomiting than bleeding.

"But, independent of this reafoning, I would imprefs upon your mind, that there may exift fome irritating matter in the ftomach, or fome exciting caufe, under the particular ftate of the nervous fyftem, which may be productive of apoplexy.

"This may be ftrongly exemplified in the cafe of fome vegetable poifons, as the drop-wort and the lauro-cerafus, &c. which have been known to produce apoplectic fymptoms, and for the removal of which, it would be an infult to your underftanding to dictate the means of relief, recommended by Dr. L.

"The free ufe I made of Dr. Kirkland's Commentary, will, I hope, induce you to perufe it attentively, as it contains many practical obfervations and ufeful diftinctions; and, I truft, you will find in this pamphlet, fubftantial fupport to what I have advanced.

"I cannot conclude without reverting to the occafion of this correfpondence, which indeed it is highly proper you fhould keep in mind. A phyfician vifits a patient juft recovered from a fit, who complains of confiderable pain in the head, occafional naufea, and flight ficknefs; there is no other mark of derangement in the fyftem; fhe is in perfect poffeffion of her underftanding, and the vital and animal actions are unimpaired. *This patient* is pronounced by the Doctor to be in a ftate of compreffed brain from a confiderable extravafation of blood.

"This is too extravagant an affertion to pafs unnoticed, and fo contrary, furely, to all authority, that medical men will not require an anfwer.

"I fear I muft have wearied you with thefe remarks upon extravafation, but my aim and endeavour have been, if poffible, to ftop the progrefs of error. The attempt is certainly to be commended, mended, however imperfectly it may be executed; for it has a tendency to promote the ſtudy and illuſtrate the doctrine of phyſiology.

"Such, I hope, may be the effects of this inveſtigation; and I aſſure you, I ſhall not regret the little trouble it has occaſioned me if it is attended with any benefit to the healing art, by inciting men of higher abilities, and more leiſure, to proſecute the Enquiry.

